# β1 integrin, ILK and mTOR regulate collagen synthesis in mechanically loaded tendon cells

**DOI:** 10.1038/s41598-020-69267-6

**Published:** 2020-07-28

**Authors:** Rouhollah Mousavizadeh, Payman Hojabrpour, Felipe Eltit, Paul C. McDonald, Shoukat Dedhar, Robert G. McCormack, Vincent Duronio, Seyed Mehdi Jafarnejad, Alex Scott

**Affiliations:** 1 Department of Physical Therapy, Centre for Hip Health and Mobility, University of British Columbia, 2635 Laurel Street, Vancouver, BC V5Z 1M9 Canada; 2 Department of Medicine, University of British Columbia, Vancouver, BC Canada; 3 Department of Materials Engineering, University of British Columbia, Vancouver, BC Canada; 4 Department of Integrative Oncology, BC Cancer Research Centre, Vancouver, BC Canada; 5 Department of Orthopaedics, University of British Columbia, Vancouver, BC Canada; 6 Patrick G. Johnston Centre for Cancer Research, Queen’s University Belfast, Belfast, Northern Ireland, UK; 7 Department of Biochemistry and Molecular Biology, University of British Columbia, Vancouver, BC Canada

**Keywords:** Molecular biology, Physiology, Molecular medicine

## Abstract

Tendons are specialized tissues composed primarily of load-responsive fibroblasts (tenocytes) embedded in a collagen-rich extracellular matrix. Habitual mechanical loading or targeted exercise causes tendon cells to increase the stiffness of the extracellular matrix; this adaptation may occur in part through collagen synthesis or remodeling. Integrins are likely to play an important role in transmitting mechanical stimuli from the extracellular matrix to tendon cells, thereby triggering cell signaling pathways which lead to adaptive regulation of mRNA translation and protein synthesis. In this study, we discovered that mechanical stimulation of integrin β1 leads to the phosphorylation of AKT, an event which required the presence of integrin-linked kinase (ILK). Repetitive stretching of tendon cells activates the AKT and mTOR pathways, which in turn regulates mRNA translation and collagen expression. These results support a model in which integrins are an upstream component of the mechanosensory cellular apparatus, regulating fundamental tendon cell functions relevant to exercise-induced adaptation and mechanotherapy.

## Introduction

Mechanical stimulation plays a pivotal role in tendon development and adaptation. Mechanical forces regulate extracellular matrix (ECM) synthesis and assembly of collagen fibers. The resident fibroblasts in tendon tissue (tenocytes) respond to mechanical stimuli by altering their gene and protein expression profiles^1^. Cell surface proteins such as integrins have been suggested to transmit physical stimuli from the tendon ECM to regulate intracellular signaling pathways and gene expression^2^. Heterodimers of α and β integrins transduce different mechanotransduction pathways, depending on the engaged isoforms and their interactions with the ECM and cytoplasmic proteins^3^. β1 integrin (also known as CD29) is a member of the subfamily of collagen-binding integrins (including α1β1, α2β1, α10β1 and α11β1) which bind to the triple-helical GFOGER sequence on collagen fibrils. The heterodimers of β1 integrin with α integrins can also bind to an RGD sequence in fibronectin and denatured collagens^4,5^. Upon binding to the ECM, integrins recruit intracellular proteins to their cytoplasmic domain including integrin-linked kinase (ILK), which plays a major role in cytoskeleton rearrangement, cell migration, proliferation and survival^6^. Importantly, ILK regulates the phosphorylation of downstream signaling effectors such as protein kinase B (PKB, also known as AKT) and glycogen synthase kinase 3 beta (GSK3β)^7,8^. However, hitherto a potential role for ILK in mechanotransduction in tendon cells has not been explored.

In certain cell types (e.g. osteoblasts), mechanical stimulation has been shown to trigger a cascade of AKT/mTOR signaling which regulates the rate of mRNA translation^9^. mTOR also plays a major role in regulation of protein synthesis in mechanically induced skeletal muscles and chondrocytes^1,2^. However, the potential role of mTOR activity and its regulation of mRNA translation in mechanically stimulated tendon cells is currently unknown.

In this study, we have investigated the role of β1 integrin and ILK in the activation of the AKT/mTOR pathway, and the role of AKT/mTOR in the subsequent expression and translation of collagen in human tendon cells. We found that these factors play a key role in the adaptive response of tendon cells to mechanical loading.

## Results

### Integrins are required for mechanical stimulation to trigger collagen gene expression and ECM organization in differentiating tendon cells

**Figure 1:**
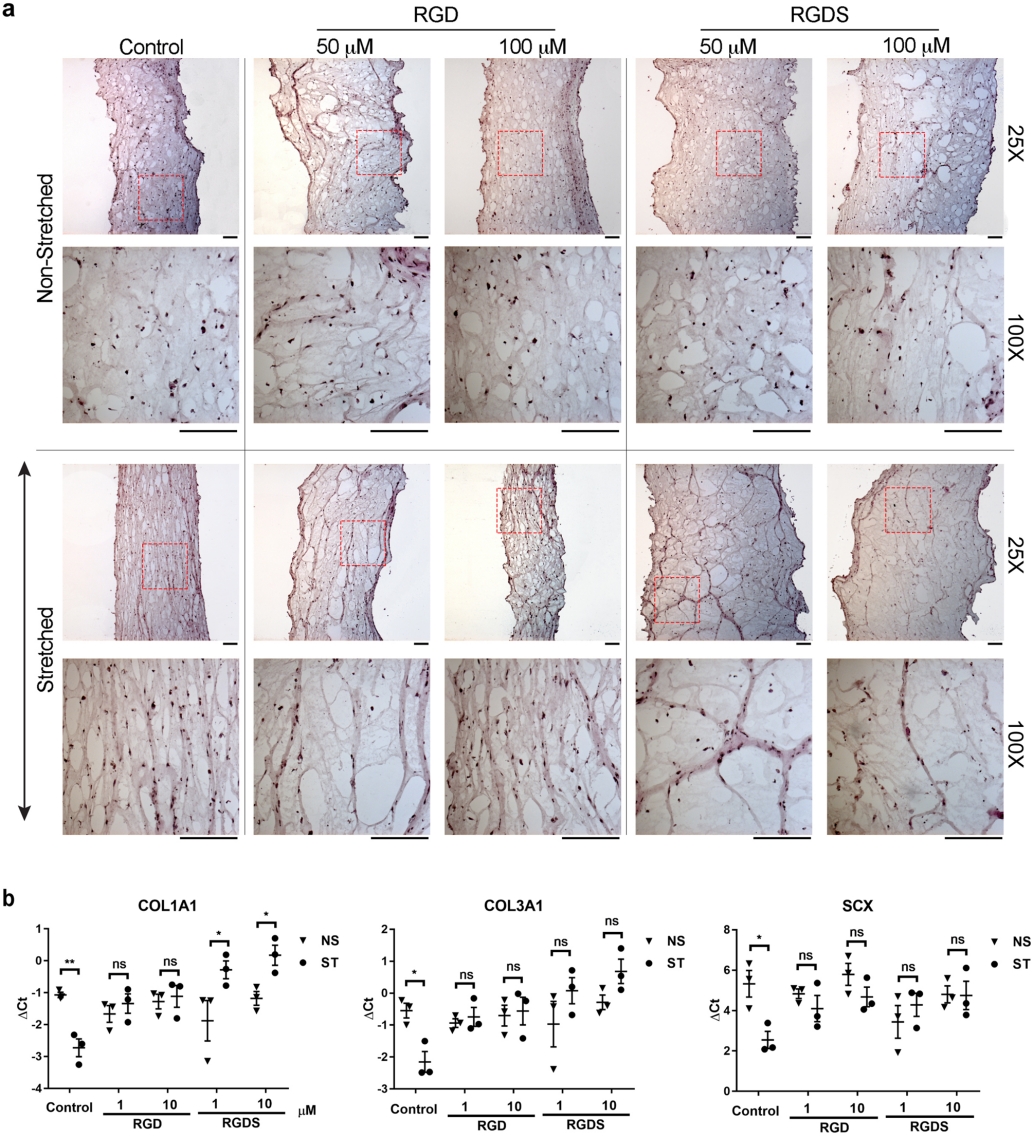
Role of integrins in modulating collagen organization and gene expression in an in vitro model of tendon differentiation. (**a**) H&E staining analysis of RGD and RGDS-induced disorganization of the ECM, which is typically oriented with the direction of stretching force. The arrow (**↕**) indicates the direction of stretching force. For each condition, the lower panel represents 4 × magnification of the selected region (dashed red box) in the corresponding upper panel. Scale bars = 250 μm (**b**) qRT-PCR analysis of regulation of gene expression in response to mechanical stimulation and treatment with RGD and RGDS. Decreasing ΔCt indicates increasing gene expression and vice-versa. Two-way ANOVA followed by Bonferroni's multiple comparison test; mean ± SE; ns P > 0.05; * P ≤ 0.05; **P ≤ 0.01; n = 3 biological replicates.

First, we explored the ability of integrins to transmit mechanical signals in an in vitro model of tendon differentiation to explore whether integrins play a role in mechanotransduction at an early stage in the life of a tendon cell. We used mesenchymal stem cells (C3H10T1/2, originally derived from mouse limb bud) as a readily available source of cells capable of undergoing tenocyte differentiation—a process which is enhanced by mechanical stretching^10^. The Tissue Train 3D Culture System (Flexcell International, USA) was used to apply uniaxial cyclic strain to the cells in a three-dimensional collagen-rich matrix, similar to in vivo tendon conditions. Inhibition of integrin attachment to the surrounding collagen matrix with RGD and RGDS peptides abrogated the normal ability of the cells and their surrounding matrix to orient longitudinally with the line of stress (Fig. 1a and Fig. S2). RGDS—which has a higher affinity to α5β1 and αvβ3 integrins compared to RGD peptide—disrupted the longitudinal orientation of the matrix (Fig. 1a and Fig. S2). Both RGD and RGDS peptides prevented mechanical strain from inducing the expression of COL1A1, COL3A1 and SCX mRNA (Fig. 1b).

### Mechanical stretching through β1 integrin stimulates AKT phosphorylation

**Figure 2:**
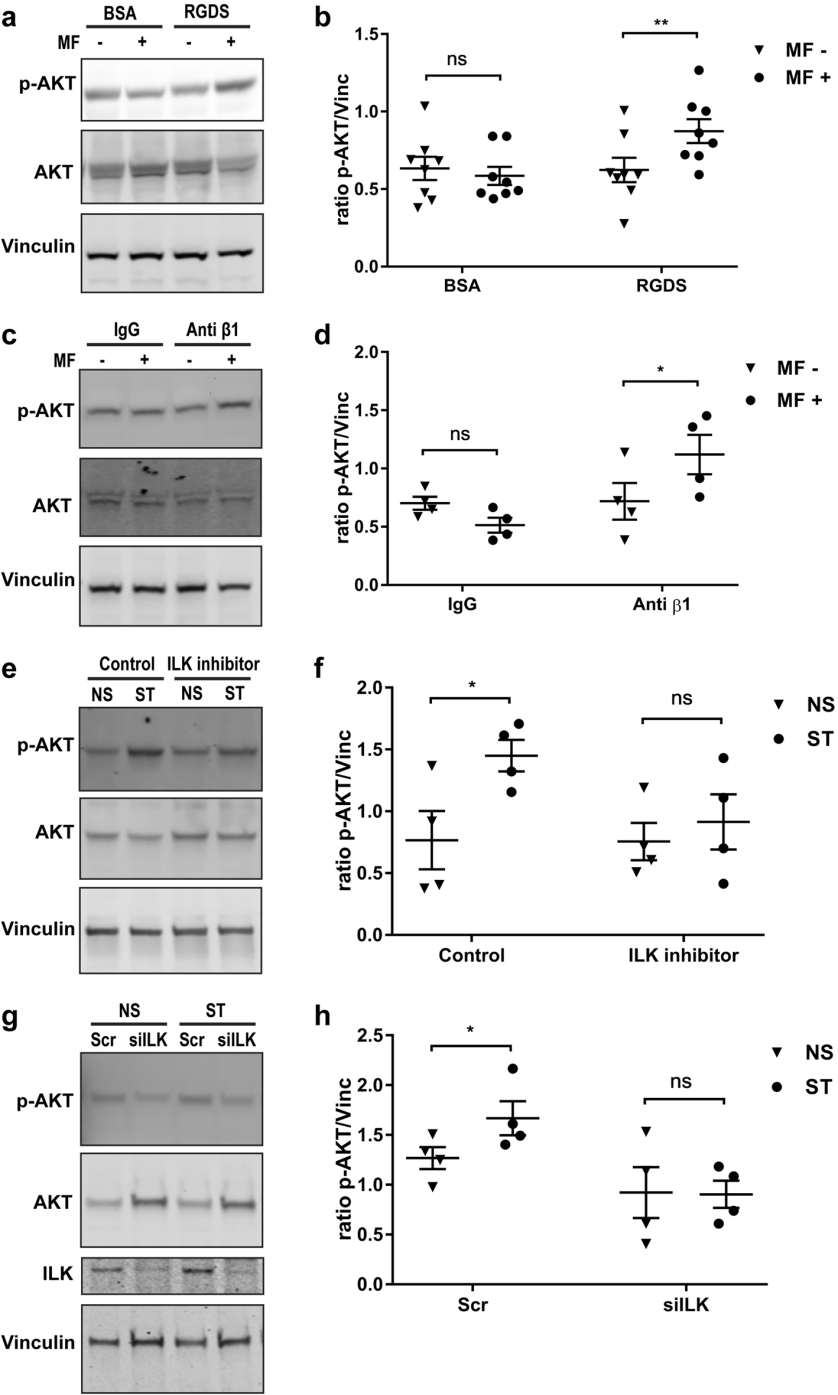
Mechanical stretching modulates AKT phosphorylation through β1 integrin and ILK. (**a**) Immunoblot of human tendon cells that were bound to RGDS-conjugated magnetic particles and exposed to one hour of oscillatory magnetic field (MF) with a frequency of 1 Hz. “- “ and “ + ” indicates without or with magnetic field, respectively. (**b**) shows the densitometry of phospho-AKT normalized to Vinculin in (**a)**. (**c**) Immunoblot of human tendon cells incubated with anti-integrin β1 conjugated magnetic particles after 1 h of oscillatory magnetic field (MF) with a frequency of 1 Hz. The immunoblots of human tendon cells incubated with ILK inhibitor QLT0267. (**d**) shows the densitometry of phospho-AKT normalized to Vinculin in (**c)**. (**e**), and siILK (**g**) indicate preventing AKT phosphorylation by mechanical stretching (ST) with Flexcell compared to the non-stretched (NS) cells. (**f)**, (**g**) show the densitometry of phospho-AKT normalized to Vinculin in (**e)** and (**g)**, respectively. Two-way ANOVA followed by Bonferroni's multiple comparisons test; mean ± SE; ns P > 0.05; * P ≤ 0.05; **P ≤ 0.01; n ≥ 4 biological replicates.

Mechanical stimulation of tendon cells in the laboratory is typically achieved by deforming the substrate on which the cells grow—however, this method could stimulate a large number of cellular structures making the study of specific cell signaling pathways extremely challenging. Therefore, in order to examine the role of integrins in mechanotransduction we targeted integrins on the surface of human tendon cells with magnetic particles conjugated to specific integrin ligands (either RGDS peptides or β1 integrin antibodies). Cyclic mechanical force was then delivered to the cells via the bound magnetic particles using controlled magnetic forces (MICA system). The resultant mechanical stretching via integrin receptors stimulated AKT phosphorylation in human tendon cells (Fig. 2a–d). Force delivered through integrins stimulated AKT to a much greater degree than through non-specific surface binding (IgG control).

### ILK regulates AKT phosphorylation during mechanical stimulation of tendon-derived stromal cells

We next examined AKT activation in tendon cells using a more traditional method of laboratory mechanical stimulation—cyclic stretching on collagen coated plates. This method has been previously used to demonstrate the regulation of collagen expression in human tendon cells by cyclic stretching, but the underlying signaling mechanisms of this response are not known^11^. Interestingly, QLT0267, a highly selective small molecule inhibitor of ILK activity^7,8^, abrogated the increased AKT phosphorylation in mechanically loaded human tendon cells (Fig. 2e). siILK had a similar effect (Fig. 2g). In both experiments, the contribution of ILK to stretch-induced AKT phosphorylation was statistically significant (Fig. 2f,h).

### Mechanical stretching induces the mTOR pathway and increases mRNA translation in human tendon cells and C3H10T1/2 cells

**Figure 3:**
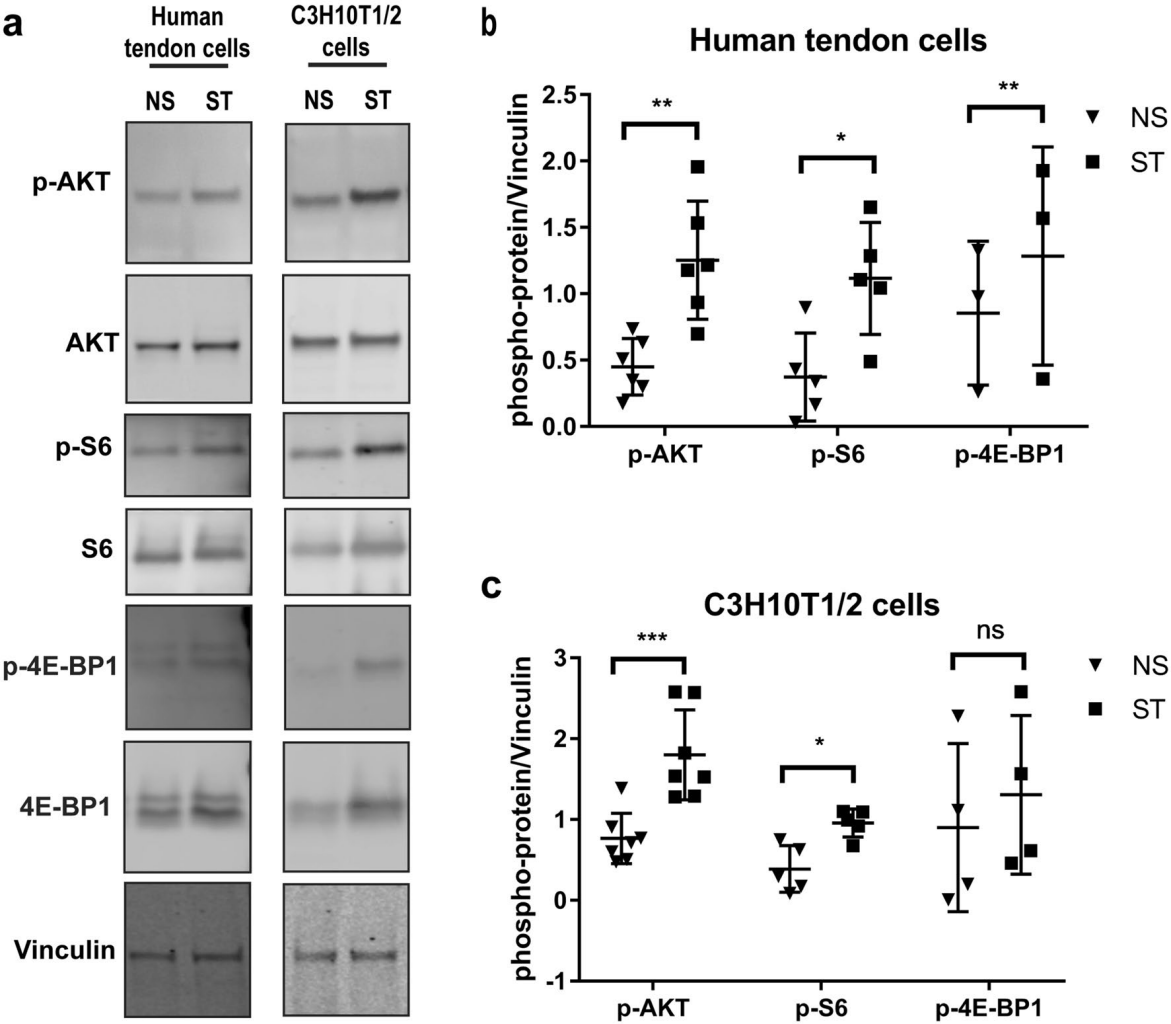
Mechanical stretching modulates mTOR signaling pathway and protein phosphorylation. (**a**) Immunoblot of protein extracts from tendon-derived stromal and C3H10T1/2 cells upon mechanical stimulation (1 h; frequency of 1 Hz). (**c**), **(d)** show the densitometry of phospho-proteins normalized to vinculin in **(a)**, **(b)**, respectively. Ratio paired t-test; mean ± SE; ns P > 0.05; * P ≤ 0.05; **P ≤ 0.01; ***P ≤ 0.001; n ≥ 3 biological replicates.

**Figure 4:**
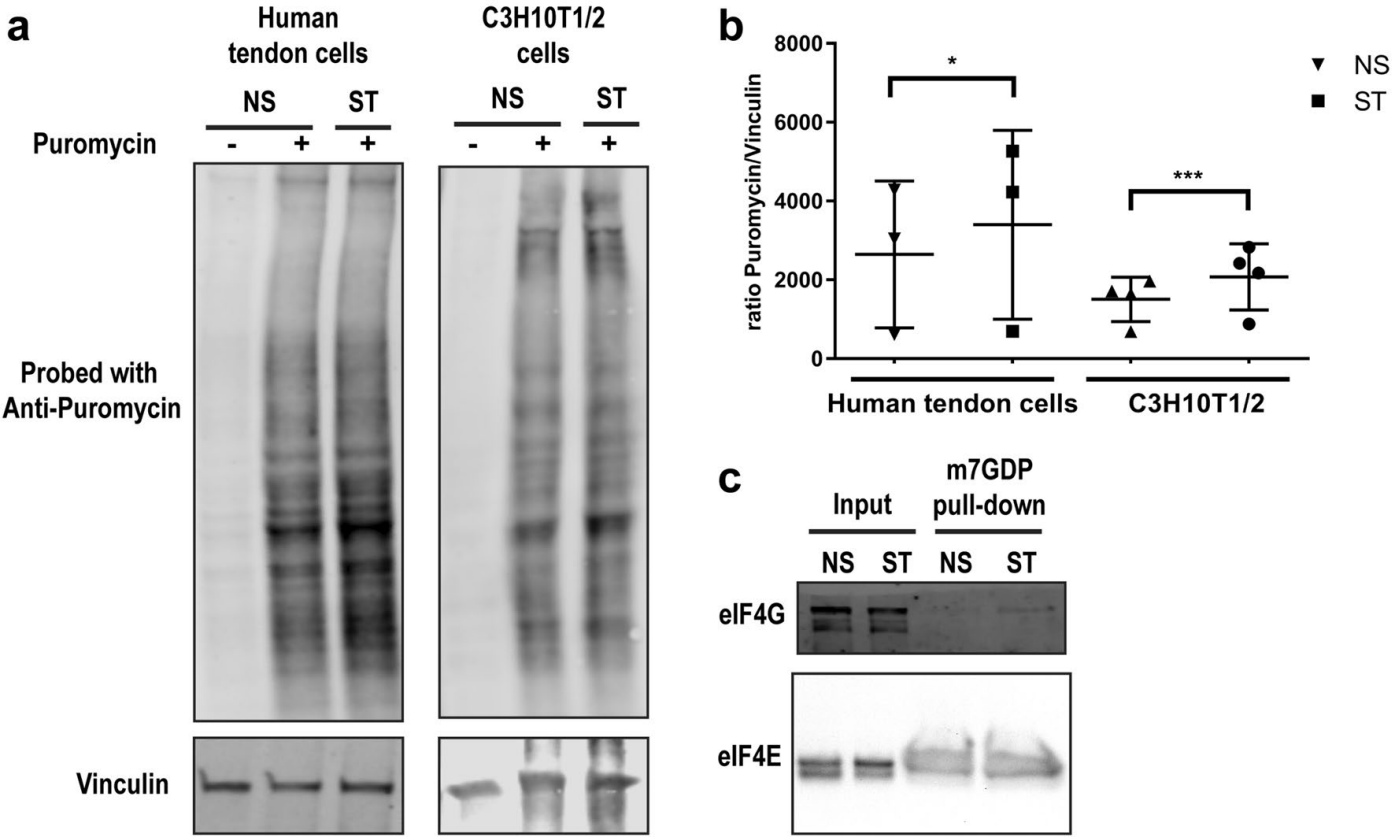
Global changes of protein synthesis and eIF4F complex formation on cap after mechanical stimulation. (**a**) Representative images of SUnSET/Western blot analysis of new protein synthesis in tendon-derived stromal and C3H10T1/2 cells after mechanical stretching. (**b**) shows the densitometry analysis of anti-puromycin blots normalized to vinculin. (**c**) Lysates from non-stimulated and stimulated (mechanical stretching) human tendon cells were subjected to m^7^GTP pull-downs and analyzed for the indicated proteins by Western blot. Ratio paired t-test; mean ± SE; ns P > 0.05; * P ≤ 0.05; ***P ≤ 0.001; n ≥ 3 biological replicates.

Given the activation of AKT by mechanical stimulation of tendon cells and considering that mTOR is one of the major signaling pathways downstream of AKT, we investigated the effect of mechanical stimulation on the mTOR pathway. After 1 h of mechanical stretching, human tendon cells and C3H10t1/2 cells consistently showed increased phosphorylation not only of AKT but also of ribosomal protein S6 (Ser240/244) and 4E-BP1 (Thr37/46), two main indicators of mTOR activity (Fig. 3a). The increased phosphorylation was significant in both human tendon cells and C3H10t1/2 cells for AKT and S6 (Fig. 3b,c). Also, 4E-BP1 was significantly hyper-phosphorylated in human tendon cells upon mechanical starching (Fig. 3b). Hyper-phosphorylation of these proteins indicates increased activity of the mTOR pathway and regulation of the mRNA translation machinery. To unravel the effects of mechanical stretching on mRNA translation and protein synthesis, we used the surface sensing of translation (SUnSET) method, which utilizes puromycin to label newly synthesized proteins followed by immunodetection using anti-puromycin antibodies. Increased puromycin labelling following mechanical stretching indicates upregulation of the rate of protein synthesis in human tendon cells and C3H10T1/2 cells (Fig. 4a,b).

eIF4F complex consists of the cap-binding eiF4E, scaffolding protein eIF4G and mRNA helicase eIF4A. eIF4F complex-mediated cap-dependent mRNA translation is the major mechanism of initiation of mRNA translation in mammalian cells^12^. mTOR regulates the formation of the eIF4F complex on mRNA cap through phosphorylation of the 4E-BP proteins (4E-BP1-3). Hyperphosphorylation of 4E-BPs result in their dissociation from eIF4E, thereby allowing the binding of eIF4G and eIF4A to eIF4E and formation of the eIF4F complex, which in turn recruits the 40S ribosomal subunit and initiates translation^13^. To test the impact of changes in activity of integrin/ILK/Akt/mTORC1 cascade on formation of the eIF4E complex as an indicator of the cap-dependent initiation of mRNA translation initiation, we performed cap pull-down assay using m^7^GTP cap analogue conjugated to Sepharose beads. Enhanced pull-down of eIF4G by cap and in this assay indicates an augmented eIF4F complex formation and increased rate of the cap-dependent mRNA translation. Our data shows that mechanical stimulation increased the amount of eIF4G which was bound to the 5′-terminal cap (Fig. 4c).

### AKT/mTOR signaling pathway regulates collagen synthesis

**Figure 5:**
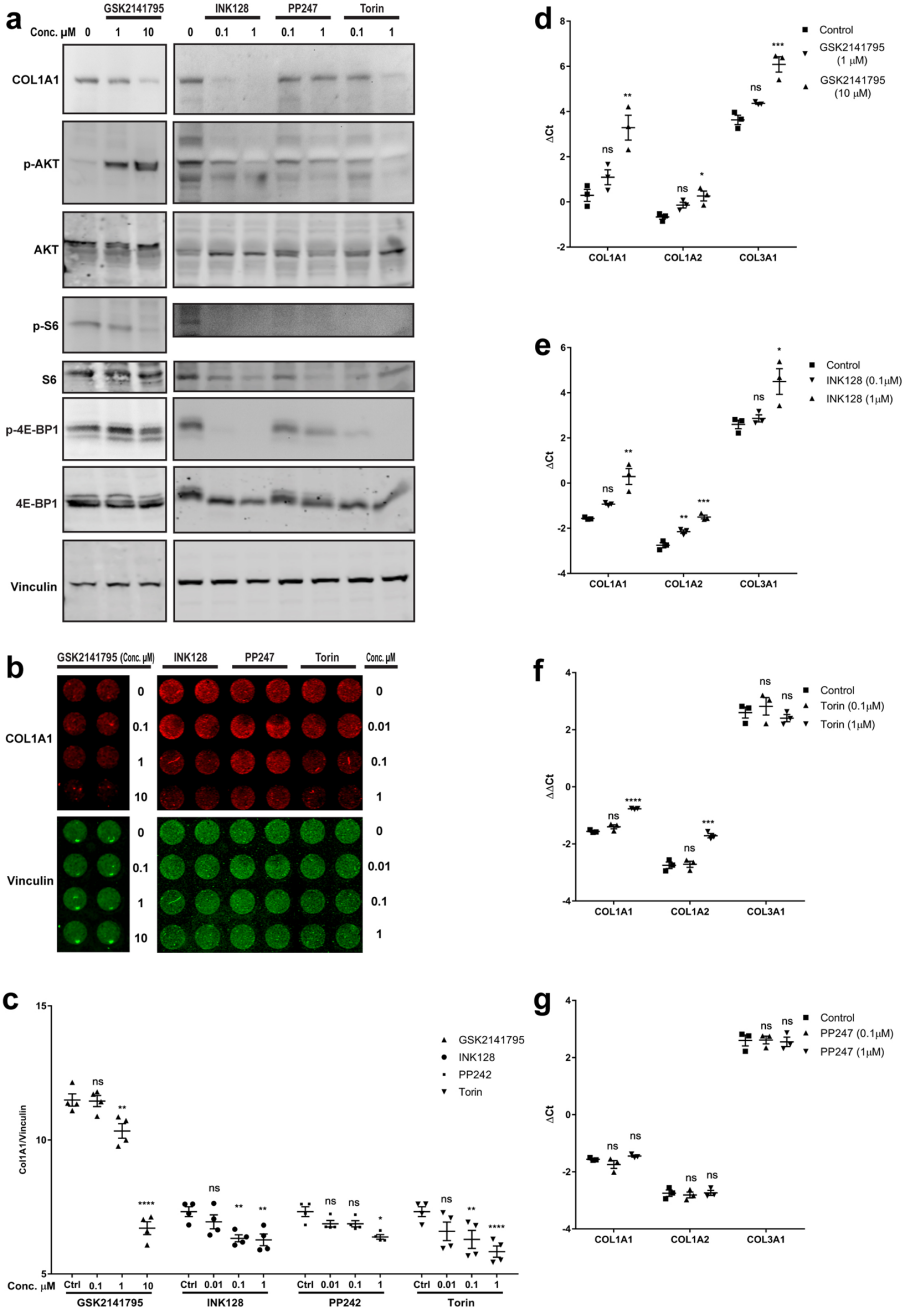
Role of mTOR pathway in regulation of collagen expression. (**a**) Immunoblot analysis of tendon-derived stromal cells after incubation with AKT inhibitor (GSK2141795) and mTOR inhibitors (INK128, PP247 and Torin) for 48 h. (**b**) In-cell western analysis of human tendon cells after 48 h and 72 h incubation with GSK2141795 and mTOR inhibitors. (**c**) Densitometry analysis of the in-cell western in **b**. (**d–g**) qRT-PCR analysis of gene expression in human tendon cells after 48 h of incubation with GSK2141795 (**d**), INK128 (**e**), Torin (**f**), and PP247 (**g**). Increasing ΔCt indicates decreasing gene expression. Two-way and one-way ANOVA followed by Bonferroni's multiple comparisons test for data of in-cell western densitometry and qPCR, respectively.; mean ± SE; ns P > 0.05; * P ≤ 0.05; ** P ≤ 0.01; *** P ≤ 0.001; **** P ≤ 0.0001; n ≥ 3 biological replicates.

We next tested if the tenocytes’ stretch-induced increase in collagen expression depends on AKT or mTOR activity. Incubation of tendon-derived stromal cells with inhibitors of AKT or mTOR for 48 h reduced the expression of collagen by tendon-derived stromal cells. Similar to mTOR inhibitors, AKT inhibitor also reduced the phosphorylation of S6K and to a lesser extent 4E-BP1; mTOR inhibitors also reduced the phosphorylation of AKT while treatment with GSK2141795, an AKT inhibitor, hyperphosphorylated AKT (Fig. 5a) likely due to a feedback mechanism that has been described previously^14,15^. At the protein level, expression of COL1A was reduced in human tendon cells that were treated with AKT and mTOR inhibitors for 48 and 72 h, respectively (Fig. 5b). Densitometry showed a significant dose-dependent reduction of COL1A1 protein after exposure to different types and concentration of AKT and mTOR inhibitors (Fig. 5c). Finally, gene expression analysis demonstrated the effect of AKT and mTOR inhibitors on collagen expression (Fig. 5d–g). AKT inhibitor and INK128 reduced the expression of COL1A1, COL1A2 and COL3A1 mRNAs. Also, while PP247 had no effect on any of these mRNA, Torin reduced the expression of COL1A1 and COL1A2 but not COL3A1 mRNA. Thus, the AKT/mTOR pathway was shown to impact collagen synthesis at both mRNA and protein levels.

## Discussion

**Figure 6:**
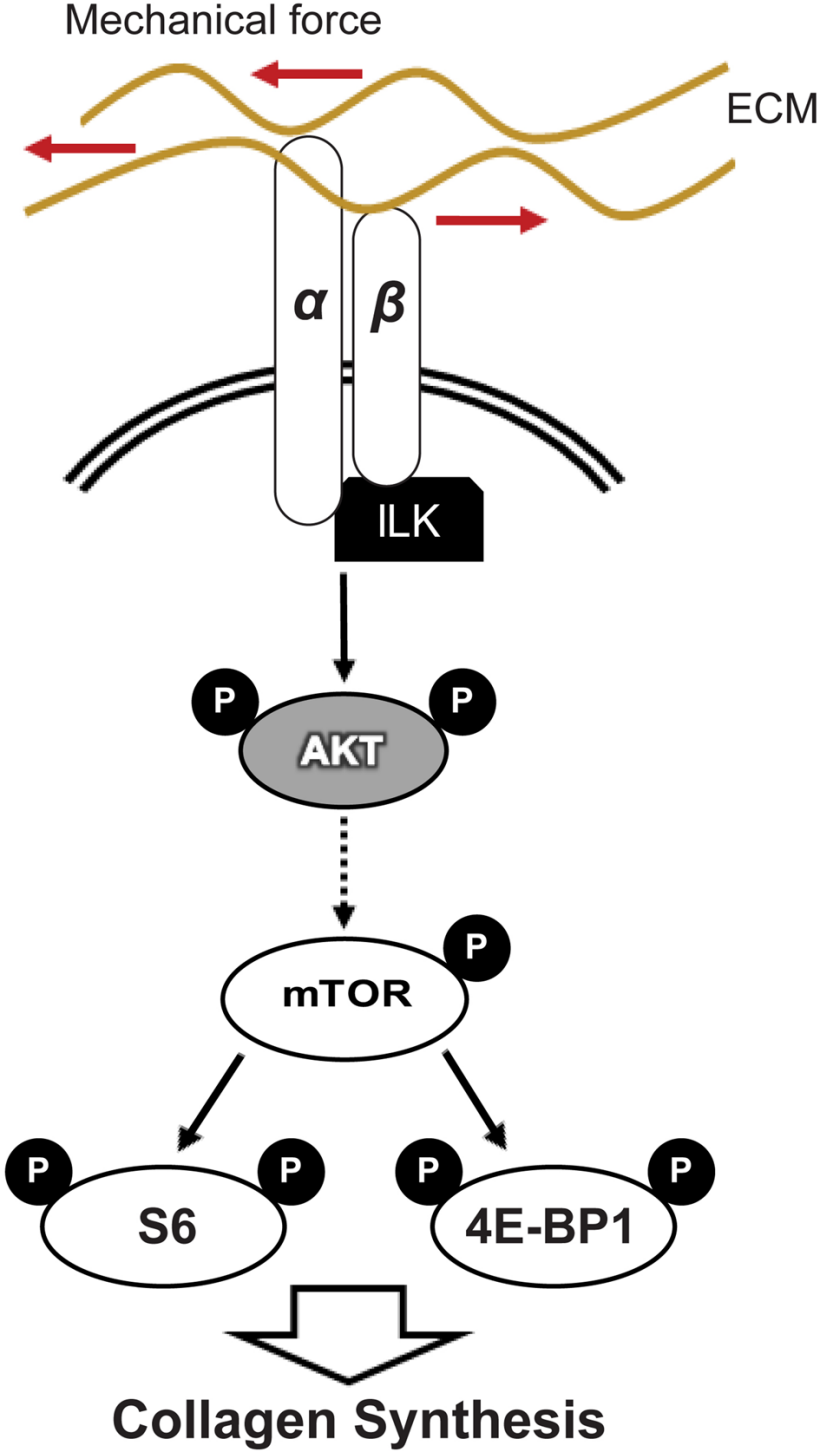
Schematic illustrating of regulation of mechanical stimulation-induced collagen expression through integrins. Integrins including integrin β1 bind to the ECM, transmit mechanical stimulation intracellularly, and activate ILK to phosphorylate AKT. AKT induces the mTOR pathway which leads to phosphorylation of S6 and 4E-BP. These events result in stimulation of collagen synthesis.

This study found that mechanical loading stimulates collagen synthesis in tendon cells by activating a signaling pathway involving β1 integrin, ILK, AKT, and mTOR. Mechanical stimulation of tendon cells exerted a potent ability to activate fundamental cellular processes such as the phosphorylation of AKT, the expression of collagen and scleraxis genes, and regulation of the rate of mRNA translation. This pathway (Fig. 6) likely contributes to the ability of tendon to adapt to physiological exercise or rehabilitation.

Previously we showed that cyclic mechanical loading promotes tenocyte differentiation and stimulates the expression of SCX and COL1A1 in mesenchymal stem cells seeded into bioartificial tendons.^10^ In this study we further demonstrated that blocking integrin receptors (with RGD and RGDS peptides) abrogated the load-induced induction of collagen and SCX. These results are in keeping with a previous study which showed that absence of integrins in Drosophila embryo dysregulated the adhesion of tendon cells to the surrounding ECM and disrupted tendon differentiation^16^. Perhaps appropriate mechanical stimulation can promote the differentiation of stem or progenitor cells toward the tendon phenotype, and if so, our data indicates that integrins may be involved in converting the mechanical cues into a differentiation signal.

Mechanical force is essential for both tendon development and adaptation^1^. Therefore, we used a 2D culture of human tendon cells subjected to cyclic strain in order to extend this study toward exploring the mechanistic role of integrins in collagen synthesis during tendon adaptation to mechanical force. Although cell behavior is different in 2D culture compared to 3D; the composition of focal adhesions in 2D and 3D matrix is quite similar^17^. Also, very thick bundles of collagen fibers in tendon tissue possibly provide a microenvironment more similar to 2D rather than 3D environment^18^. Our data indicates that one hour of 10% biaxial mechanical loading induces AKT phosphorylation and mTOR activity in human hamstring tendon cells and mesenchymal stem cells and. Other studies have shown increased activity of AKT in fibroblasts, osteoblasts and mesenchymal stem cells by mechanical stress^19–23^. Uniaxial mechanical stimulation induces AKT activity in 2D and 3D culture of tendon-derived stem cells, and this activity promotes tenogenic differentiation^24^. We also found that β1 integrin and ILK are crucial for AKT activity induced by mechanical stimulation. In particular, our finding that QLT-0267 inhibited stretch-induced AKT phosphorylation suggests the importance of ILK in regulating this process. Interestingly, ILK also plays a role in cardiac function and has recently been shown to regulate AKT phosphorylation by vertebrate heart cells to control contractility^25^. ILK is an adaptor protein which links β1 integrin and intracellular protein complex to regulate localization and orientation of cytoskeleton protein^26,27^. β1 integrin and ILK sense mechanical signals from collagen type I matrices to regulate AKT phosphorylation and cell survival in fibroblast cells^28^. Thus, we provide evidence in support of previous observations pertaining to the involvement of β1 integrin, ILK and AKT in mechanotransduction.

ILK also interacts with Rictor, a critical component of mTORC2, to regulate AKT phosphorylation^29^. AKT activation can subsequently activate the mTORC1 protein complex which leads to phosphorylation of ribosomal protein S6 and 4E-BPs, which play important roles in the regulation of mRNA translation^30^. We found that mechanical stretching increased the phosphorylation of S6 and 4E-BP1. Although we did not compare different loads of mechanical stretching in regulation of mTOR pathway, Chen et al. shows high elongation mechanical stretching (12%) leads to increased P70S6K (a direct substrate of mTORC1 and kinase for ribosomal protein S6) and subsequent RUNX2 expression in rat tendon cells while low elongation (4%) has opposite effects^31^. Additionally, we revealed increased association of eIF4G protein with the mRNA 5´-terminal cap after mechanical stretching. Phosphorylation of 4E-BP leads to its dissociation from eIF4E which increases the binding of eIF4G to eIF4E to form eIF4F protein complex on mRNA 5´ cap structure. This event is crucial for recruitment of ribosome to mRNAs and initiation of mRNA translation^32^. Further molecular studies could focus on identifying the newly translated mRNA after mechanical stimulation of tendon cells at a transcriptome-wide scale.

Our study strongly suggests a role for mTOR/AKT signaling in collagen synthesis, and are in keeping with previous reports that examined this regulatory mechanism. We showed that AKT and mTOR inhibitors reduced phosphorylation of 4E-BP1 and ribosomal protein S6. mTOR inhibitors also decreased AKT phosphorylation, while GSK2141795 a potent ATP-competitive inhibitor of AKT kinase activity regardless of AKT phosphorylation status increased AKT phosphorylation, likely due to a feedback mechanism that has been described previously^14,15^. Walker et al. showed phosphorylation of both S6K1 and 4E-BP1 contribute to collagen I expression in fibrotic mesenchymal stem cells^33^. We showed inhibition of mTOR by INK128, PP242 and Torin reduce collagen synthesis. GSK2141795 also has a similar effect. Several other studies (in fibroblasts and mesangial cells) have also shown a role of the AKT pathway in regulating collagen synthesis^34–37^. Activation of the AKT/mTOR pathway was also recently shown to play a crucial role in tendon differentiation and collagen production in mesenchymal stem cells^38^. A loss- and gain-of-function study in mouse by Lim et al. showed that mTOR signaling is essential for tendon maturation, playing a key role in the assembly of tendon matrix. Their results indicate that loss of mTORC1 signaling disrupts assembly of collagen fibers and decreases the expression of ECM genes including collagen^39^. Regulation of collagen synthesis is vital in the maintenance and adaptation of tendon tissues, which are predominantly composed of collagen fibers^40^. Several factors control collagen synthesis at the transcriptional and translational levels. For example, La ribonucleoprotein domain family member 6 (LARP6) regulate collagen mRNA translation, and NFATc4 binds to collagen promoter to activate COL1A1 mRNA expression^41,42^. Dysregulation of collagen turnover in pathological tendons disturbs the integrity and mechanical properties of tissue^43^. Several underlying conditions such as aging, diabetes, hypercholesterolemia and obesity increase risk of tendon injuries^44,45^. Also, some metabolic factors such as oxidized LDL, and medications (e.g. corticosteroids) impair collagen synthesis which may contribute to tendon injuries^46^. Although the role of collagen synthesis and AKT/mTOR pathway in tendon injuries is not fully understood, several studies suggest the implication of this pathway in tendon and musculoskeletal diseases^47^. Perhaps a lack of physiologic mechanical loads after rotator cuff tendon tear, for example, may contribute to decreased activity of AKT/mTOR pathway, with a resulting loss of mechanotransduction signaling.

In conclusion, this study demonstrates a role for integrins in the mechanical activation of the AKT/mTOR pathway in human tendon cells. These results, along with previous work by other groups, support a mechanism where mechanical stretching activates the AKT/mTOR pathway via β1 integrin; AKT/mTOR then induce the phosphorylation of 4E-BP and S6 to regulate collagen expression (Fig. 6). Further exploration of this pathway may assist in the development of mechanotherapy strategies for tendon injuries.

## Material and methods

### Cell culture

Tendon-derived stromal cells were isolated and cultured as previously described^48^. Healthy human hamstring (semitendinosis) tendons (excess anterior cruciate ligament autograft material) were acquired from male patients age between 27–39. The tissues were minced and digested by Collagenase D. The digested materials were cultured after several washes with PBS. The protocol was approved by University of British Columbia Clinical Research Ethics Board (certificate number H10-00220), and each donor provided written informed consent. All participants had experienced a complete rupture of the anterior cruciate ligament but were otherwise healthy. The average age of participants was 32.71 (SD ± 5.93), and 8 males were included. All methods in this study were performed in accordance with the relevant guidelines and regulations.

A mesenchymal stem cell line (C3H10T1/2 cells, originally derived from embryonic mouse tissue and obtained through a generous gift from Dr Underhill at University of British Columbia) were grown in high glucose Dulbecco’s modified Eagle’s medium (DMEM) supplemented with 10% fetal bovine serum, 2 mM l-glutamine, 100 units/ml penicillin, and 100 µg/ml streptomycin in a humidified incubator containing 5% CO_2_ at 37 °C. Enzyme Free Cell Dissociation Solution (EMD Millipore Corporation, USA, #S-014-B) was used to detach and passage the cells. To inhibit the ILK pathway, QLT0267, a highly selective inhibitor of ILK activity^7,8^, was added to cell culture media at a concentration of 10 µM immediately prior to mechanical stretching of the cells. Cells were transfected with siRNA against ILK with siLentFect Lipid Reagent (BIO-RAD, USA, #1703362) according to the manufacturer’s instructions. Media were replaced after 24 h of transfection and cells were subjected to mechanical stretching after 2 days without changing the media. mTOR inhibitors that were used include INK128 (Selleck Chemicals, USA, # S2811,) Torin 1 (Sigma-Aldrich, USA, #475991) and PP242 (Tocris Bioscience, USA, #4257). GSK2141795 (Selleck Chemicals, USA, #S7492), an AKT inhibitor, was used to block the pathway.

### Bio artificial tendon (BAT)

BATs were prepared as previously described^10^. RGD (Abcam, USA, #ab142698) and RGDS (Tocris Bioscience, USA, # 3498) were added to the neutralized mixture of Purecol EZ Gel (Sigma-Aldrich, USA, # 5074), 5 × low glucose DMEM, fetal bovine serum and C3H10T1/2 cells before the mixture was pipetted into each well of an untreated Tissue Train plate (Flexcell International Corp., USA, #TT-5001U). The gels were set in an incubator for two hours and BATs were covered with 2 ml complete media including RGD or RGDS.

### Mechanical stimulation

BATs were exposed to uniaxial cyclic strain (0.1 Hz frequency, 10% strain) with 10 s rest insertion for 1,000 cycles per day for up to 10 days. Mechanical stretching was also applied to two-dimensionally cultured cells in BioFlex plates (Flexcell International Corp., Hillsborough NC, USA) as previously described^48^. Cells were seeded on six well BioFlex Culture Plates coated with collagen type I (Flexcell International Corp., USA, #BF-3001C) 2 days prior to applying mechanical stretching. Plates were transferred to the Flexcell station without changing media and with minimal disturbance. Equibiaxial cyclic strain (1 Hz frequency, 10% strain) was applied using the FX-4000T Tension Plus Unit (Flexcell International Corp., Hillsborough NC, USA).

### Integrin stimulation

In order to apply mechanical force to integrins and β1 integrin, the Magnetic Force Bioreactor (MFB) technique was used as previously described^49^. Carboxyl FerroMagnetic particles (Spherotech, USA, #CFM-40-10) were covalently conjugated with BSA, rat IgG isotype control (Invitrogen, USA, # 02-9602), RGDS peptide (Tocris Bioscience, USA, # 3498), or β1 integrin antibody (DSHB, USA, #AIIB2) using EDC (Pierce, USA, #PG82079) according to the manual. Cells were seeded in six well plates. After 2 days, the media were replaced with serum free DMEM including about 40,000 of the conjugated particles per ml and the cells were incubated for 2 h. The cells were washed three times with PBS. Complete medium was added, and the cells were incubated overnight. The plates were placed in a controlled magnetic field produced by Magnetic Force Bioreactor (MFB) (MICA Biosystems, Stoke-on-Trent, UK). The experimental plates received an oscillatory magnetic field for 1 h at 1 Hz, and the control plates were incubated in the absence of the magnetic field. The specificity of conjugated particles with ITGB1 antibody was assessed by comparing the microscopic micrographs of the cells bound to the particles conjugated with IgG iso control antibody and ITGB1 antibody (Figure S1a). Also, bound particles in protein lysates were counted in a hemocytometer and normalized to total protein concentration (Figure S1b).

### Histology

BATs were embedded in O.C.T. compound and flash frozen with liquid nitrogen. The specimens were sectioned into 5 µm and fixed in 10% buffered formalin followed by H&E (hematoxylin and eosin) staining. The stained sections were micrographed using a digital camera (AxioCam ICm 1, Zeiss, Germany) attached to a microscope (Axio Observer.A1, Zeiss, Germany).

### Analysis of BATs micrographs

In order to observe and quantify if the experimental conditions affect the orientation of the extracellular matrix in the bioartificial tendons, we quantified the orientation of histological features.^50^ For that purpose, digital images were converted into 8-bit files and analyzed with ImageJ software using the “particle analyze” function. The images were converted to binary files, with a standard threshold. Elements > 10 pixels and roundness > 0.1 within the BATs, were identified, their major axis determined, and their angle relative to the major axis of the BAT determined and plotted on a histogram. A minimum of 500 elements were measured in a representative tissue section from each condition.

### Western blot

Total protein was harvested in lysis buffer (50 mM Tris–Cl, pH 7.7; 1% Triton X-100; 10% glycerol; 100 mM NaCl, 2.5 mM EDTA, 10 mM NaF) supplemented with a protease inhibitor cocktail (Roche, Germany, #04693124001) and PhosSTOP (Sigma-Aldrich, USA, # 4906845001). The cell lysates were homogenized by sonication on ice. The homogenates were spun at 13,000 *g* for 10 min. The protein concentrations of the supernatants were measured using the BCA Protein Assay Kit (Pierce, USA, #23225). 20 µg of total protein samples were boiled in loading buffer and resolved by electrophoresis in Novex 4–20% Tris–Glycine Mini Gel (Thermo Fisher Scientific, USA, #XP04205BOX). The resolved proteins were transferred to a 0.45 µm nitrocellulose membrane (Biorad, Germany, #162-0115) in cold transfer buffer (25 mM Tris, 192 mM glycine, 20% methanol) with a wet transfer apparatus. The membranes were blocked with 5% BSA in Tris-buffered saline with 0.05% Tween 20 (TBST), and probed with Phospho-AKT (Ser473) (Cell Signaling Technology, USA, #4060S), AKT (BD Biosciences, USA, #610861), Phospho-S6 Ser 240/244 (Cell Signaling Technology, USA, #2215), S6 (Cell Signaling Technology, USA, #2217), Phospho-4E-BP1 Thr37/46 (Cell Signaling Technology, USA, #2855) , 4E-BP1 (Cell Signaling Technology, #9644) and Vinculin (Sigma-Aldrich, USA, #v9131) and COL1A1 (Cell Signaling Technology, USA, #84336) antibodies in TBST overnight at 4 °C, followed by 3 × 10 min TBST washes and labeling with IRDye 800, IRDye 680 and HRP-conjugated secondary antibodies in TBST. SuperSignal West Femto Chemiluminescent Substrate (Thermo Fisher Scientific, USA, #PI34095) were used to detect HRP-conjugated antibodies.

### Equipment and settings

The immunoblots were visualized using the Odyssey CLx Imaging System (LI-COR, USA) and G:BOX Chemi XT4 Gel Documentation System (Syngene, UK) using the default settings for IR- and HRP-conjugated antibodies, respectively. Exposure, brightness, and contrast were uniformly adjusted on all samples on each blot using the pertinent software of the image detection system. Images were exported as TIF file and ImageJ was used for western blot quantification of protein bands. Adobe Illustrator was used to compile the images.

### In cell western assay

The expression of collagen, Type I, alpha 1 protein was measured in human tendon cells with an in-cell western method. Human tendon cells were seeded in a 96-well microplate (Corning, USA, #C3603) with a density of 10,000 cells per well. mTOR inhibitors (INK128, PP242 and Torin) were added after 2 days and the cells were incubated for 72 h. Cells were fixed with 4% formalin following permeabilization with Triton × 100 and blocking with Blocker Casein in TBS (Thermo Fisher Scientific, USA, #37532). The cells were incubated with Anti-Collagen I (Abcam, USA, ab34710) and Vinculin (Sigma-Aldrich, USA, #v9131) antibodies following incubation with IRDye 680 anti-rabbit and IRDye 800 anti-mouse secondary antibodies. The plate was scanned using the Odyssey CLx Imaging System (LI-COR, USA).

### Surface sensing of translation (SUnSET) assay

Changes in protein synthesis after exposure to mechanical stimulation was measured by a modified method of SUnSET assay^51^. Cells were treated with 2 µg/ml puromycin during 1 h of mechanical stimulation. Control cells were treated with 100 µg/ml cycloheximide for 5 min to stop mRNA translation prior to adding puromycin. Total protein was harvested after mechanical stretching and newly synthesized proteins were visualized by anti-puromycin antibody (Sigma-Aldrich, USA, #MABE343) by western blot.

### Cap pull-down assay using m^7^GTP-sepharose

Cells were lysed in 4 volumes of lysis buffer (50 mM MOPS/KOH (pH:7.4), 100 mM NaCl, 50 mM NaF 2 mM EDTA, 2 mM EGTA, 1% NP40, 1% Na-DOC + add 7 mM BME, protease inhibitors and 1 mM Na3VO4 or phosphatase inhibitor cocktail 1) on ice for 15 min with occasional vortexing. After clearing the lysate (16100 x *g*/10 min at 4 °C), 50 µl of m^7^GTP-Sepharose 4B beads (Jena Biosciences, Germany) was incubated with 500 µg of cell lysates for 30 min at 4 °C, washed five times (5 min each) with the same buffer, and eluted with 0.2 mM m^7^GTP for 15 min at 4 °C. Eluted proteins were subjected to SDS/PAGE followed by western blotting using eIF4G (Cell Signaling Technology, USA, # 2469) and eIF4E (BD Bioscience, USA, #610270) antibodies.

### Gene expression analysis by qRT-PCR

Total RNA was extracted using GeneJET RNA Purification Kit (Thermo Fisher Scientific, USA, # K0732) and the concentration were measured using NanoDrop 2000 (Thermo Fisher Scientific, USA). 1 µg total RNA was reverse transcribed to cDNA with a High Capacity cDNA Reverse Transcription Kit (Applied Biosystems, USA, #4368814) according to the manuals. The primers (Table S1) were designed by PrimerQuest Tool and synthesized by Integrated DNA Technologies. qPCR was run using 7500 Fast Real-Time PCR System (Applied Biosystems, USA) and Luna Universal qPCR Master Mix (New England Biolabs, USA, #M3003) according to the manufacturer’s instructions. Each cDNA samples were run as duplicates using 10 ng cDNA for each qPCR reaction. The PCR reaction was performed using following steps: denaturation at 95 °C for 1 min, followed by 40 cycles at 95 °C for 15 s and 60 °C for 30 s. The specificity of the PCR amplification products was verified by melting curve analysis. GAPDH was set as a reference, and ΔCt (= Ct _Target_ – Ct _GAPDH_) values were calculated with Ct mean of each target gene. ΔCt values were plotted and used for statistical analysis.

### Statistical analysis

The densitometry of immunoblots was evaluated with ImageJ software. The data of densitometry (ratio phospho-protein/vinculin) was analyzed with ANOVA followed by Bonferroni's multiple comparison test or ratio paired T-test. Gene expression data (ΔCt) values were examined by ANOVA followed by Bonferroni's multiple comparison test. The in cell western assay signals were captured using Image Studio Lite (LI-COR, USA) and the data were examined by ANOVA followed by Bonferroni's multiple comparison test. The statistical analyses were carried out using GraphPad Prism. The number of biological replicates is reported in figure captions. P values of less than 0.05 were regarded as statistically significant.

## Supplementary information

Supplementary Information.
