# Supplementary material for: β1 integrin, ILK and mTOR regulate collagen synthesis in mechanically loaded tendon cells
Source: Sci Rep. 2020 Jul 28;10:12644. doi: 10.1038/s41598-020-69267-6 (PMC7387456; doi:10.1038/s41598-020-69267-6)

Supplementary table S 1: List of primers used for real time qPCR.

Gene	Forward primer	Reverse primer
Mouse Col1a1	GCTTGAAGACCTATGTGGGTATAA	GGTGGAGAAAGGAGCAGAAA
Mouse Col3a1	AGATAAGGGTGAAGGTGGTTCC	CCTGGTTCACCATTCTGTCC
Mouse Scx 72bp	GAACACCCAGCCCAAACAGA	CTGTCACGGTCTTTGCTCAAC
Mouse Gapdh	TCACCACCATGGAGAAGGC	GCTAAGCAGTTGGTGGTGCA
Human Col1a1	CACCAATCACCTGCGGTACAGAA	CAGATCACGTCATCGCACAAC
Human Col1a2	AGA GTG GAG CAG TGG TTA CTA	GAT ACA GGT TTC GCC AGT AGA G
Human Col3a1	AATCAGGTAGACCCGGACGA	TTCGTCCATCGAAGCCTCTG
Human Gapdh	TCTTTTGCGTCGCCAGCCGAG	TGACCAGGCGCCCAATACGAC

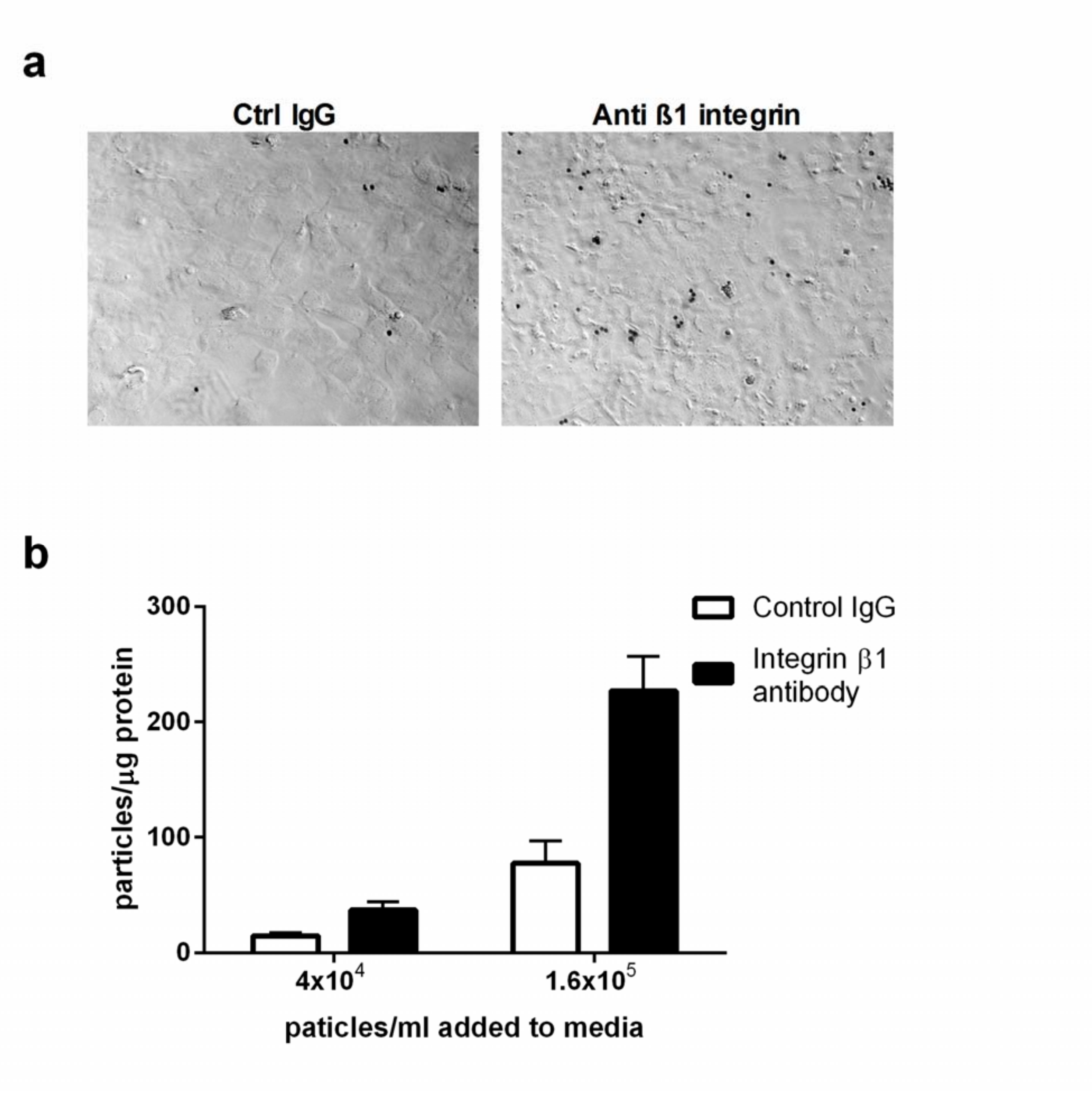

Supplementary figure S1: Specificity of conjugated particles with ITGB1 antibody

a) The microscopic micrographs of the cells bound to the particles conjugated with IgG iso control antibody and ITGB1 antibody.
b) The number of bound particles in protein lysates which were normalized to total protein concentration.

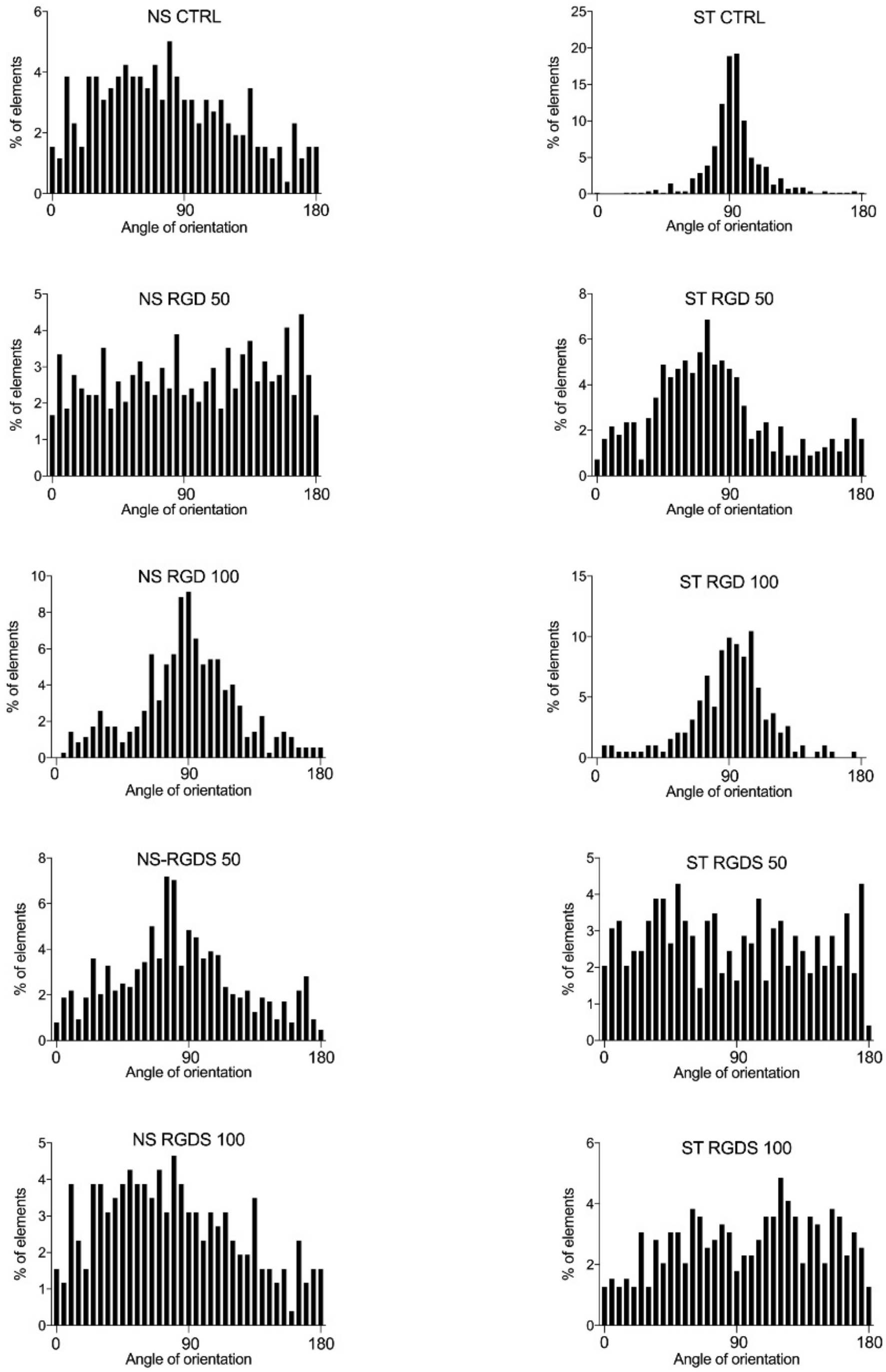

Supplementary figure S2: Plot of alignment of structural features of BATs in Figure 2a

The sum of elements oriented within 5 degrees intervals are represented in a single bar. 90 degrees represents alignment on the major axis of the bioartificial tendon. Left column corresponds to non-stretched condition, right column corresponds to stretched condition.

Supplementary figure S3: Uncropped images of blots used in main figures. Number of the corresponding figure is shown above the panels. Regions used for figures are boxed. Positions of the molecular mass markers are shown as kDa. (*) indicates residual signal from previous blotting.

Figure 2(a)

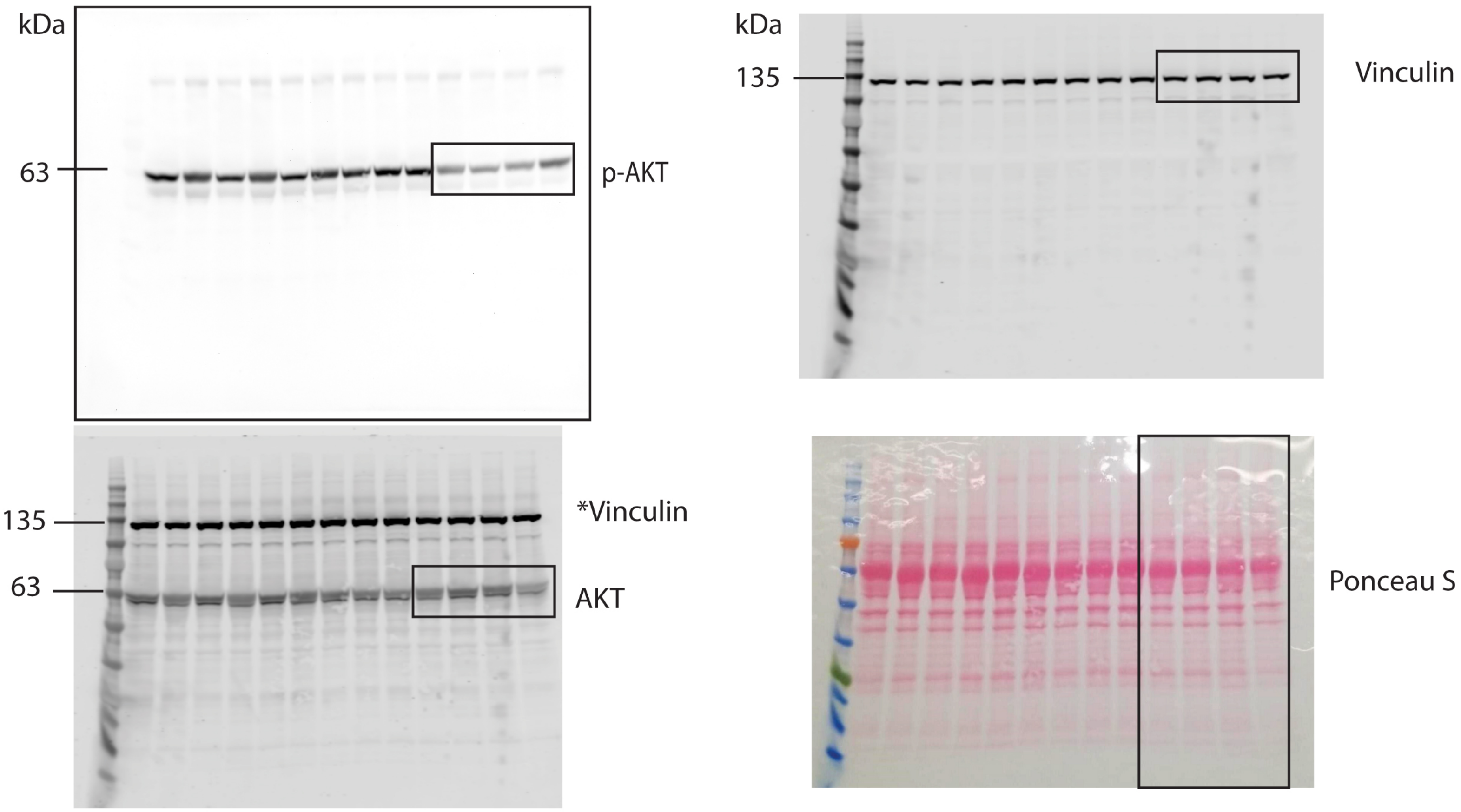

Figure 2(c)

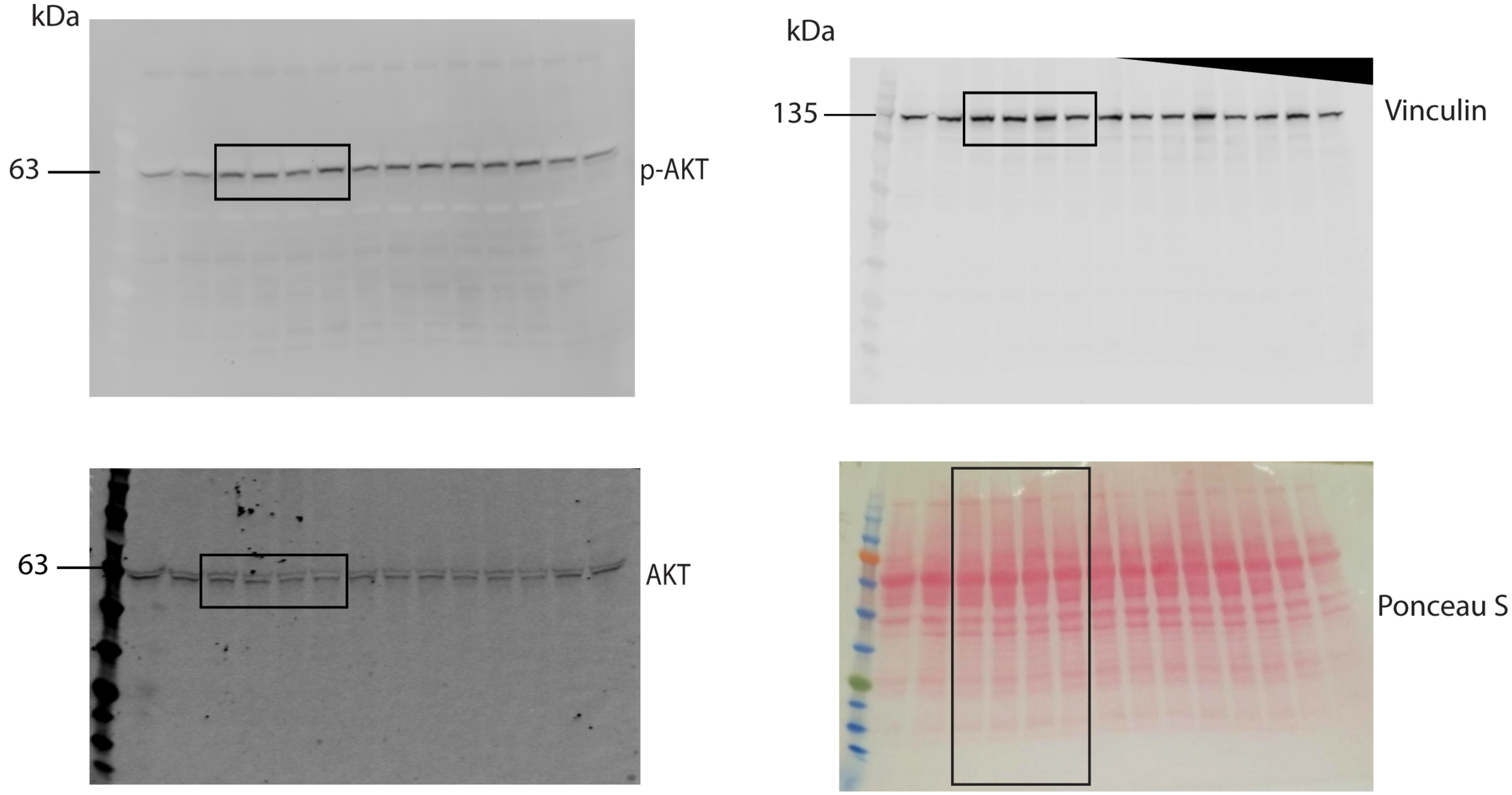

Figure 2(e)

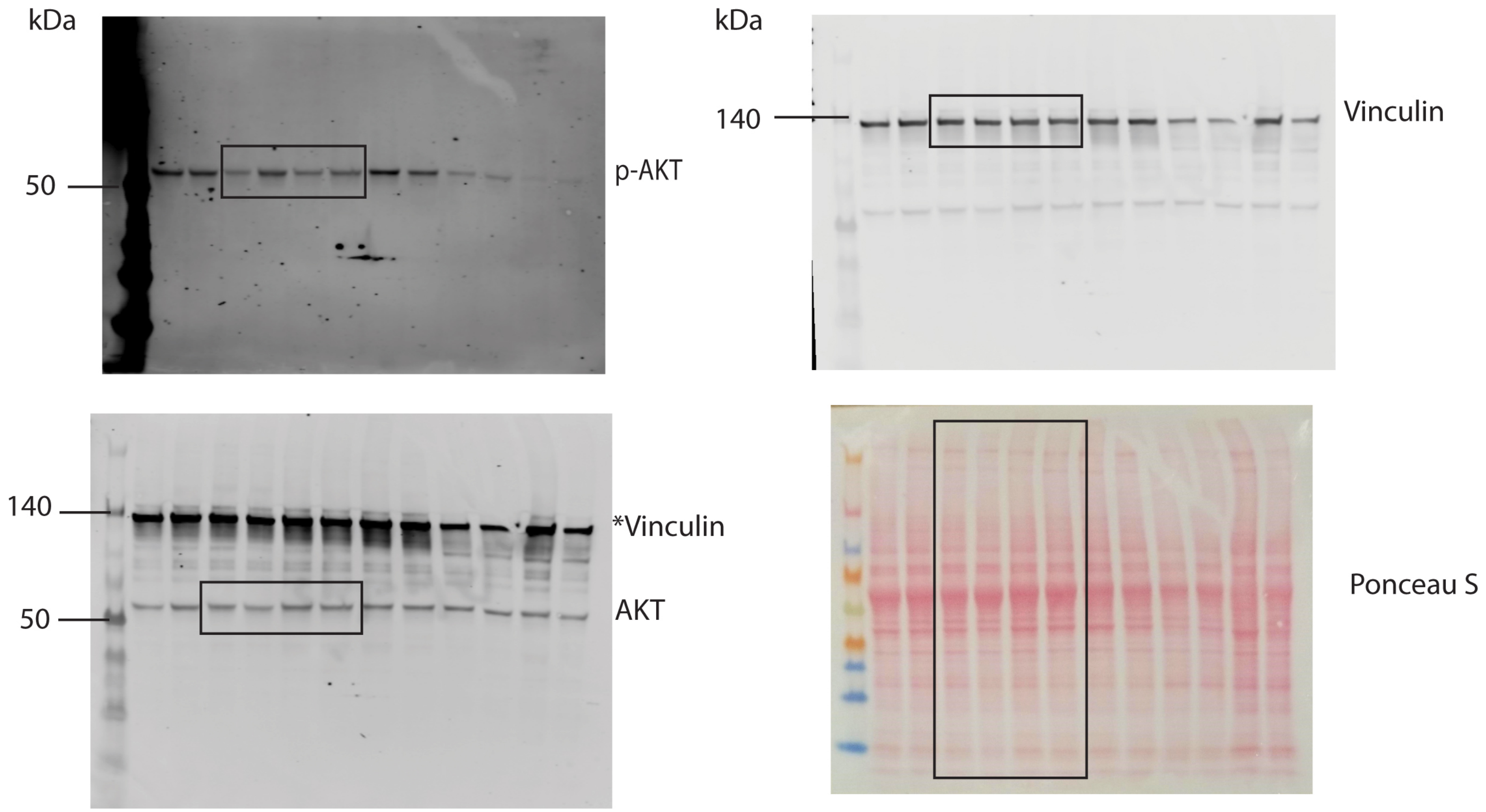

Figure 2(g)

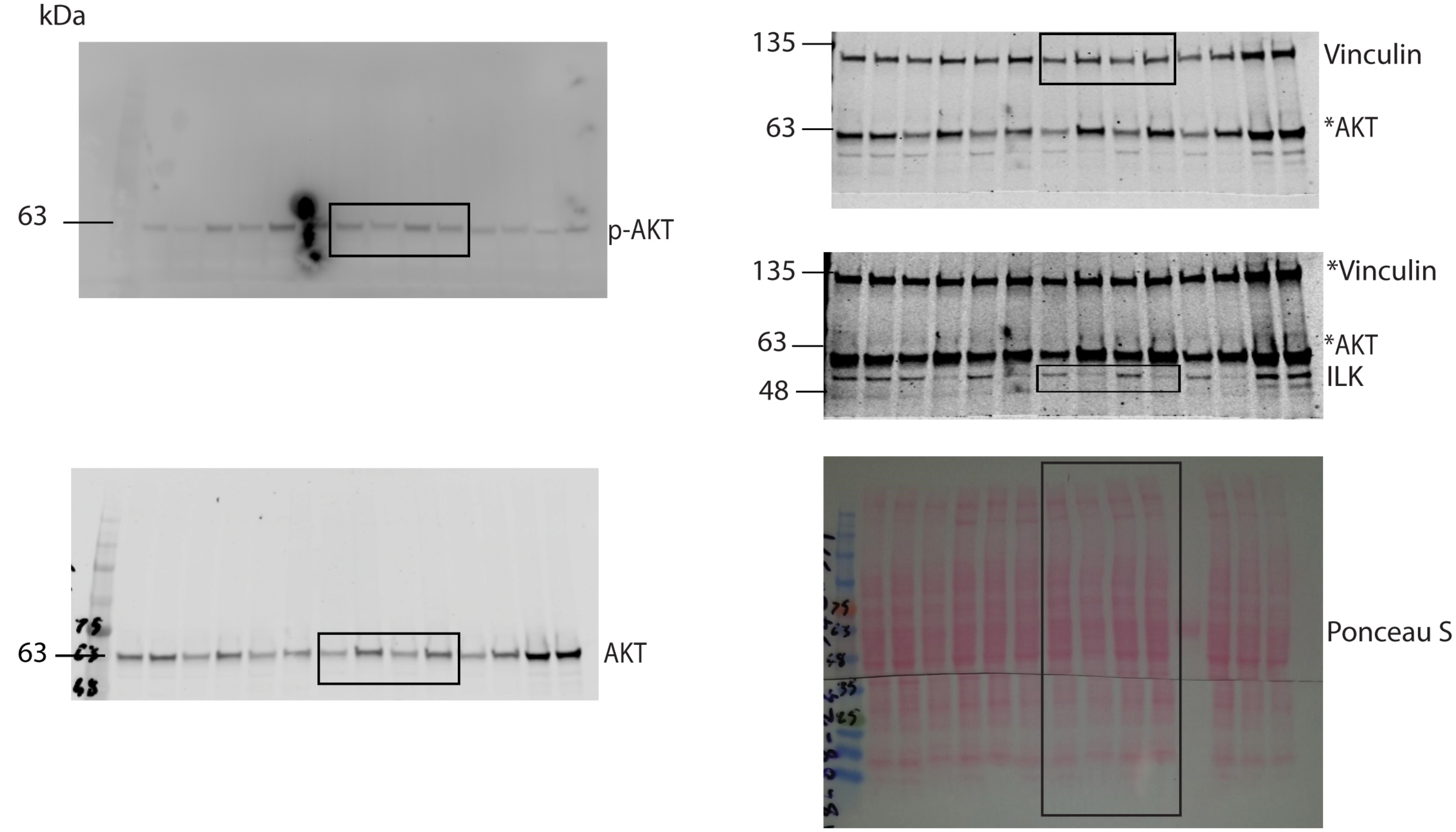

Ponceau S staining was done after cutting the mebrane for simultaneous probing.

Figure 3 (human tnedon cells)

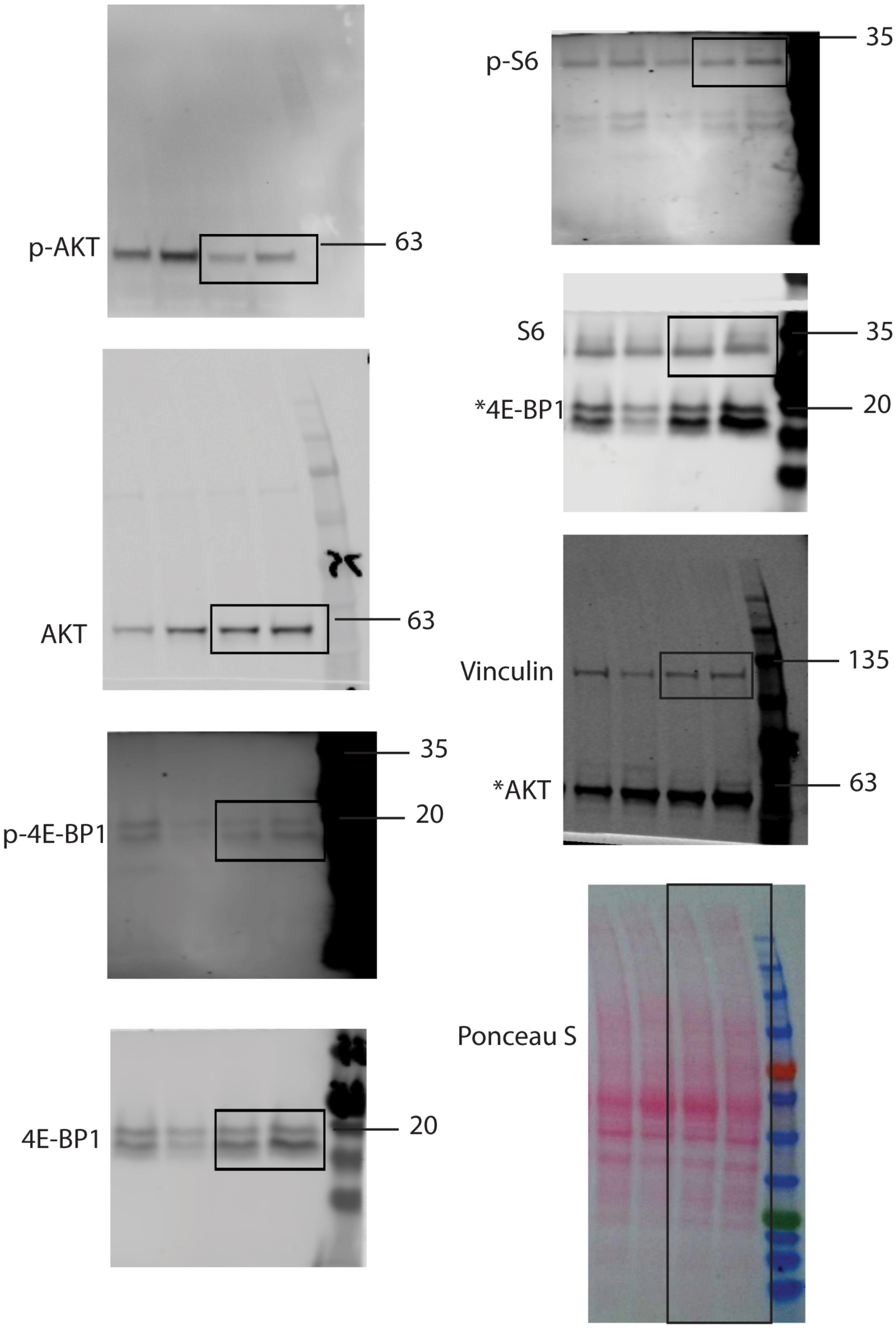

Figure 4(a) Human tendon cells

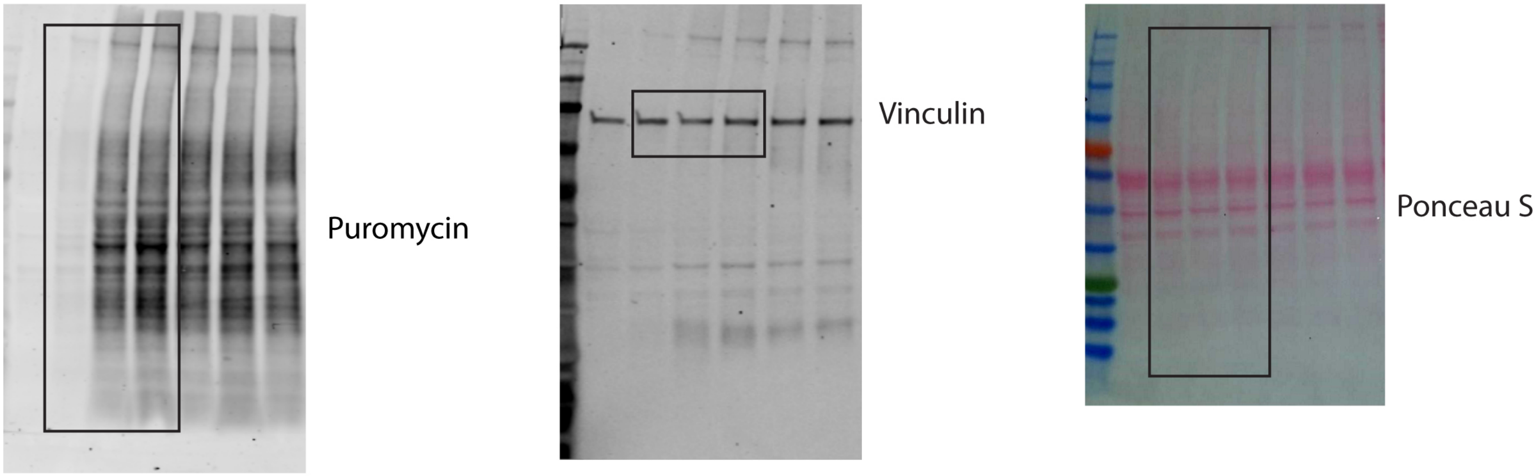

Figure 4(a) C3H10T1/2 cells

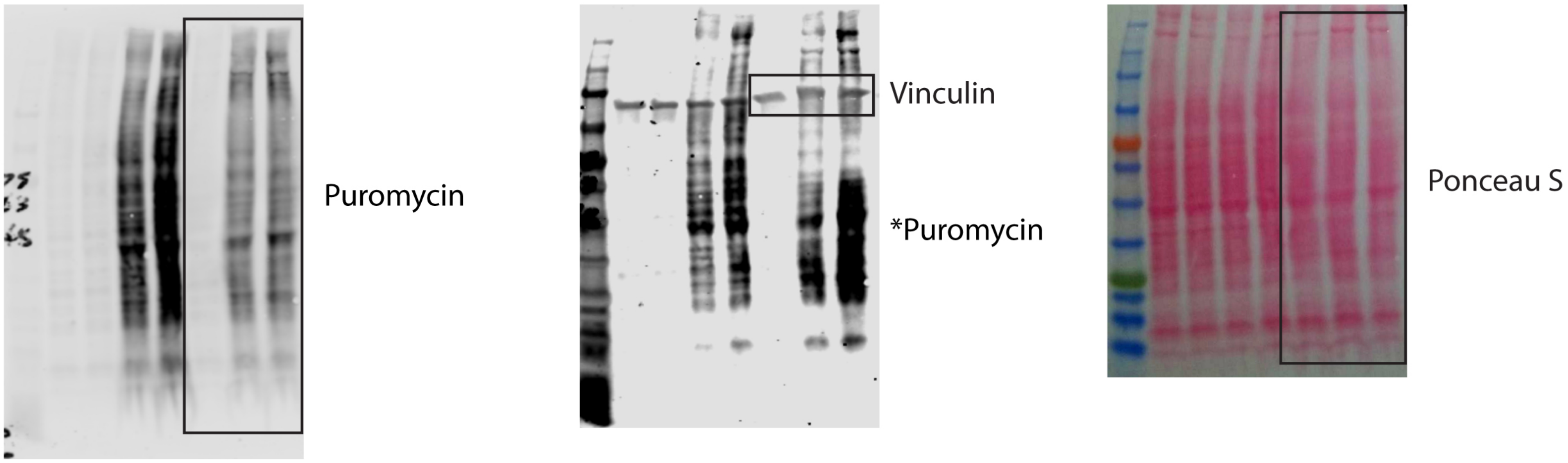

Figure 4(c)

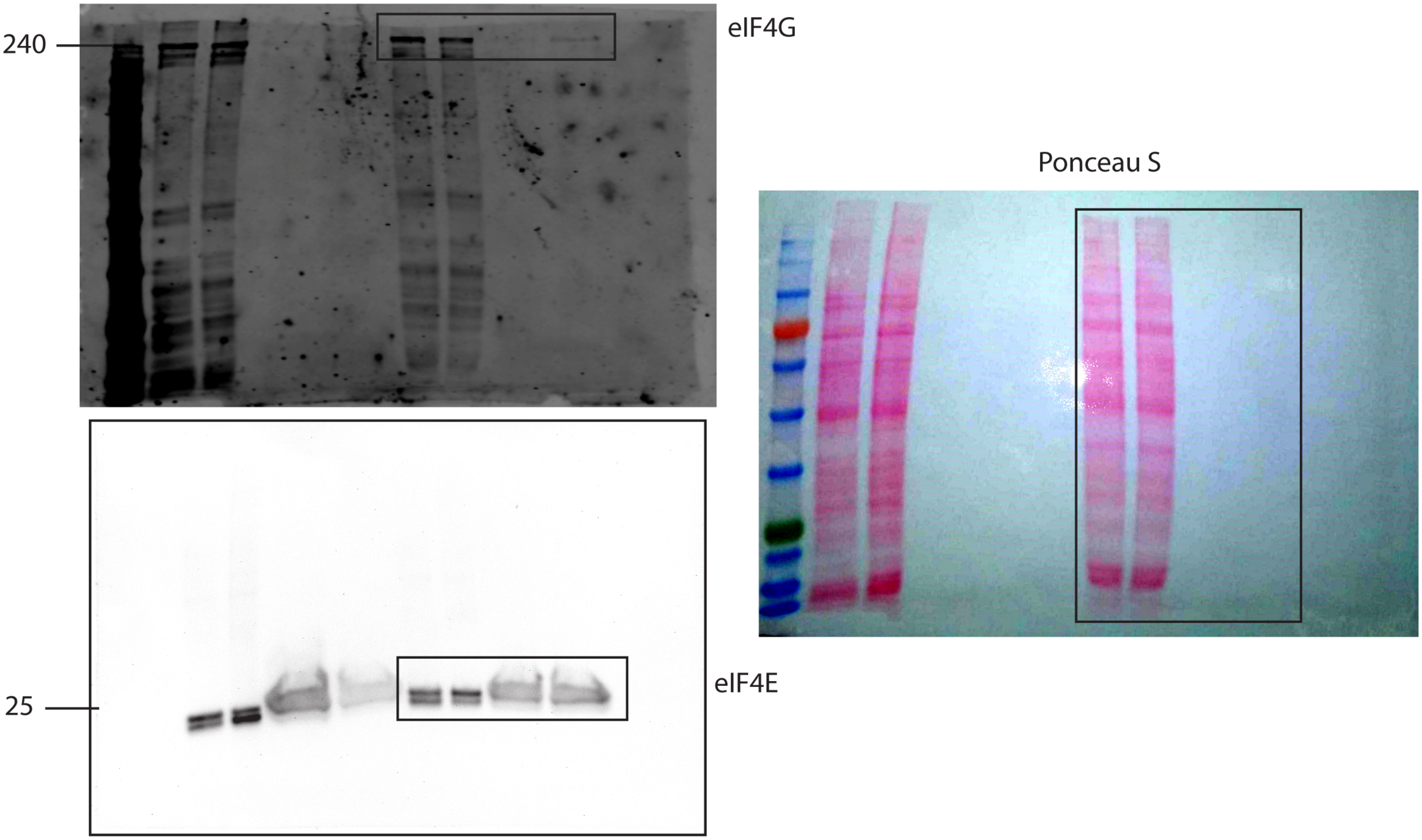

Figure 3 (C3H10T1/2)

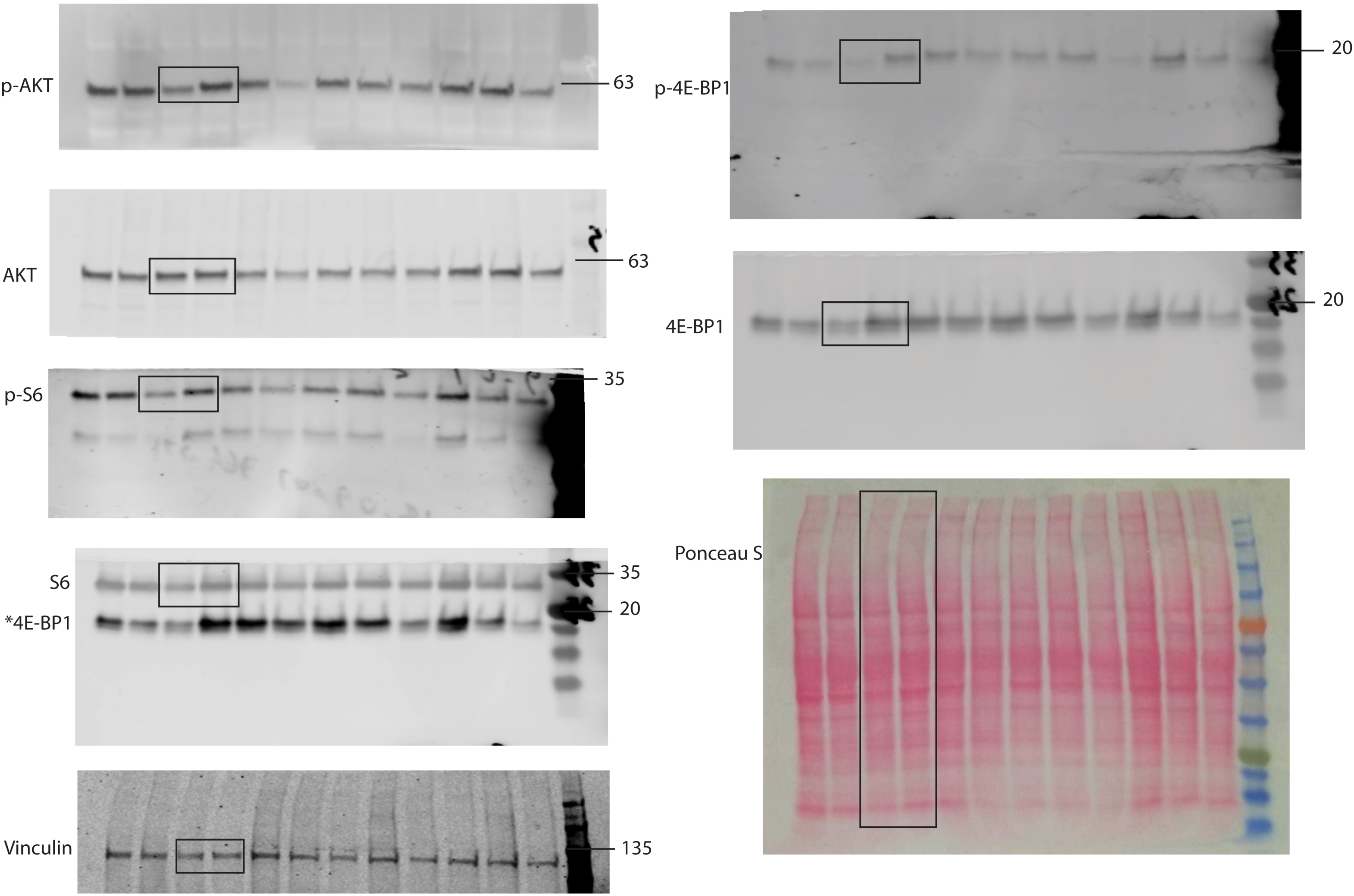

Figure 5(a) (GSK2141795)

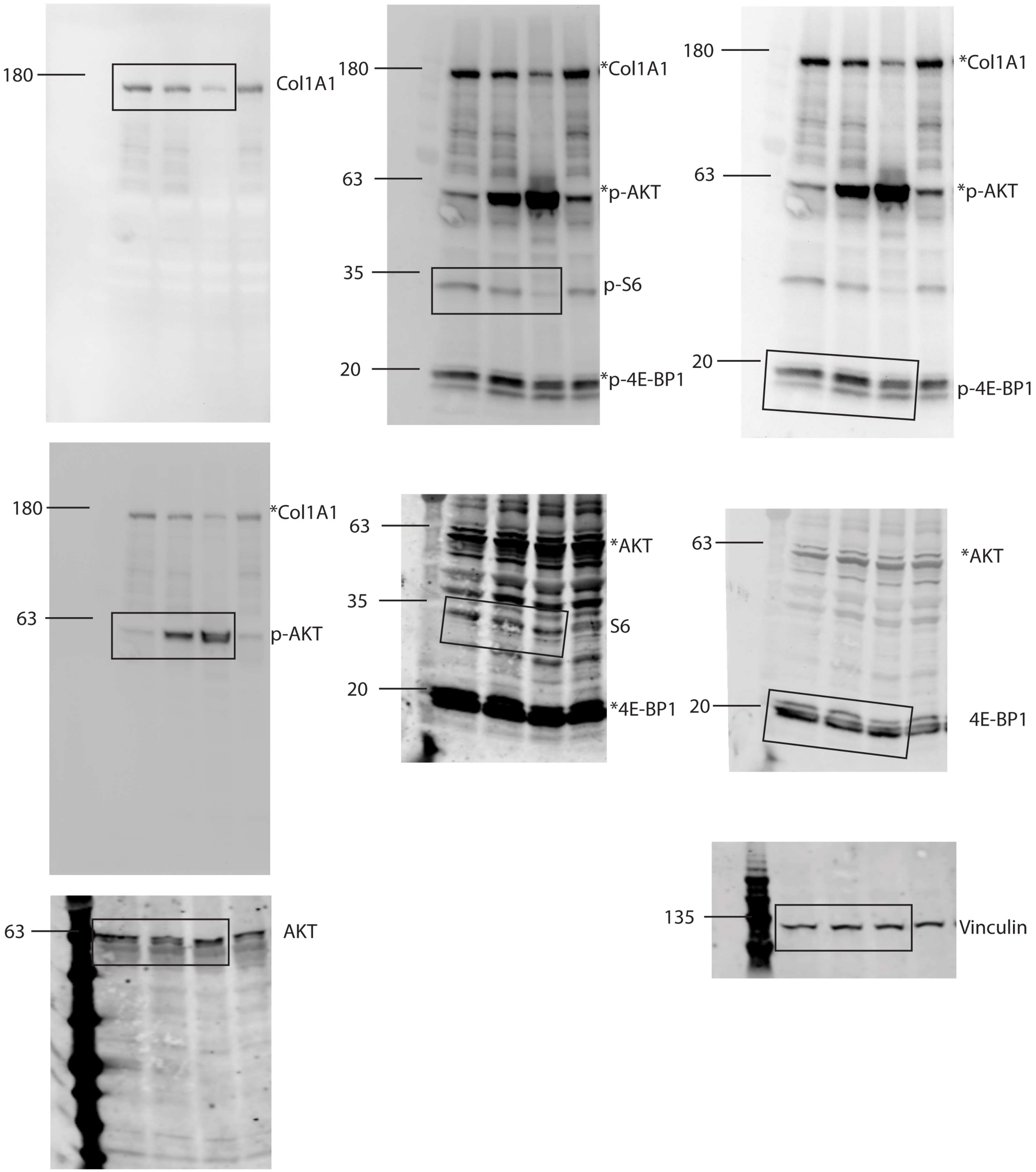

Figure 5a (mTOR inhibitors)

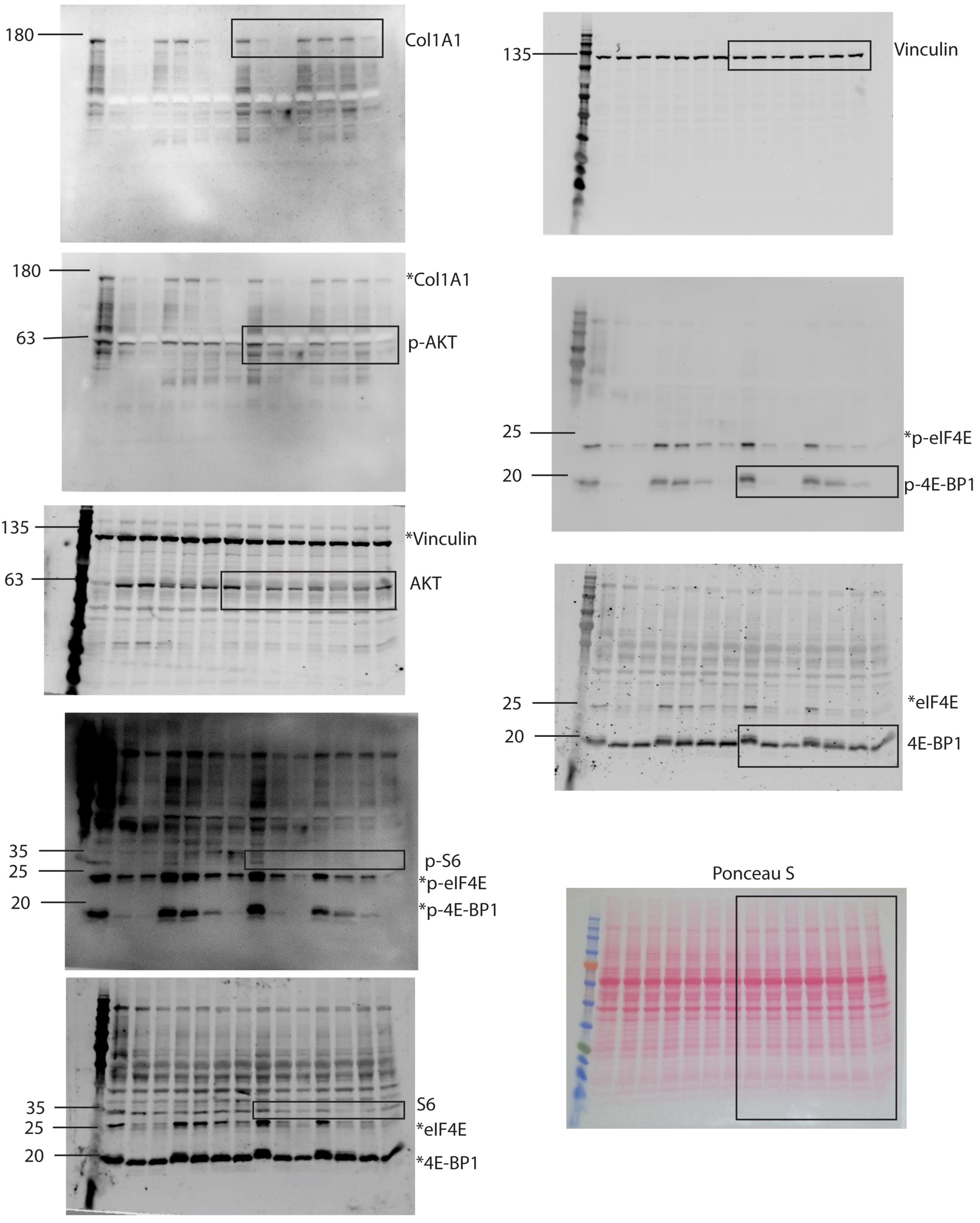

Supplement: Supplementary file 1 — Supplementary Information. [file 41598_2020_69267_MOESM1_ESM.pdf]
